# Rituximab for the Management of an Australian Cohort of Treatment Refractory Mucous Membrane Pemphigoid

**DOI:** 10.1111/ajd.14523

**Published:** 2025-05-16

**Authors:** Bronte Jeffrey, Mark Schifter, Elizabeth Arena, Emily Sullivan, Stephanie Rose, David Joo, David Campbell, Suzanne Culican, David McDonald, Ming Wei Lin

**Affiliations:** ^1^ Department of Clinical Immunology Westmead Hospital Sydney Australia; ^2^ St Vincent's Clinical School University of New South Wales Sydney Australia; ^3^ Department of Immunopathology, NSWHP‐ICPMR Westmead Hospital Sydney Australia; ^4^ Faculty of Medicine and Health University of Sydney Sydney Australia; ^5^ Department of Oral Medicine Westmead Hospital Sydney Australia; ^6^ Centre for Immunology and Allergy Research Westmead Institute of Medical Research Sydney Australia

**Keywords:** biomarkers, bullous, oral, pemphigoid, rituximab

## Abstract

**Background:**

Mucous membrane pemphigoid (MMP) has a broad range of clinical manifestations, from relatively benign self‐limiting oral lesions to significant scarring (cicatrizing) of the oral, nasal and ocular tissues with severe functional impairment and morbidity. European Guidelines recommend rituximab as only second‐ or third‐line therapy, based on the extent/severity of the disease; however, there are no established clinical or serological markers that are predictive of severe disease warranting the use of agents such as rituximab.

**Methods:**

Retrospective cross‐sectional cohort study of patients who met the following criteria: (1) biopsy confirmed MMP; (2) required a steroid‐sparing immunosuppressant therapy, that is, mycophenolate and/or rituximab and (3) at least 6 months of clinical monitoring. The primary end point was complete or partial remission.

**Results:**

Of the 45 patients who met the criteria, 12 (27%) had sustained remission with mycophenolate. Thirty‐three (73%) patients had either relapsed or were refractory to mycophenolate and, therefore, were treated with rituximab. Of those who received rituximab, 97% achieved a complete remission after a single course (1 g given intravenously on Days 1 and 14), but 24% needed repeat treatment. The detection rates of key circulating antibodies, namely skin basement membrane antibodies (SBMA), BP180/230, collagen VII and laminin 332, were low and did not identify those patients refractory to mycophenolate. Adverse reactions, including infectious complications, were minimal in both patient groups.

**Conclusion:**

In our study of mostly localised mucosal MMP patients, there was an excellent response to a single course of treatment with rituximab, with durable remission and no major adverse complications.

## 1 Introduction

Mucous membrane pemphigoid (MMP) is not a single disease; rather, it is a heterogeneous group of chronic, subepithelial, blistering diseases that primarily affect mucosal surfaces [1]. MMP was first differentiated from pemphigus and bullous pemphigoid by Walter Lever in 1953, who named the disease ‘benign MMP’. Despite this initial terminology, patients can have life‐threatening or severely disabling, organ‐threatening manifestations. This is because one of the hallmarks of MMP is that when the bulla rupture, severe ulceration occurs down to the level of the connective tissues, which subsequently heals with scarring and with repeated cycles of bulla/blister eruption and breaking; this, results in severe cicatrisation [1]. Consequently, there is a range of disease, from mild desquamative gingivitis to severe manifestations such as conjunctival scarring, leading to blindness.

**FIGURE 1:**
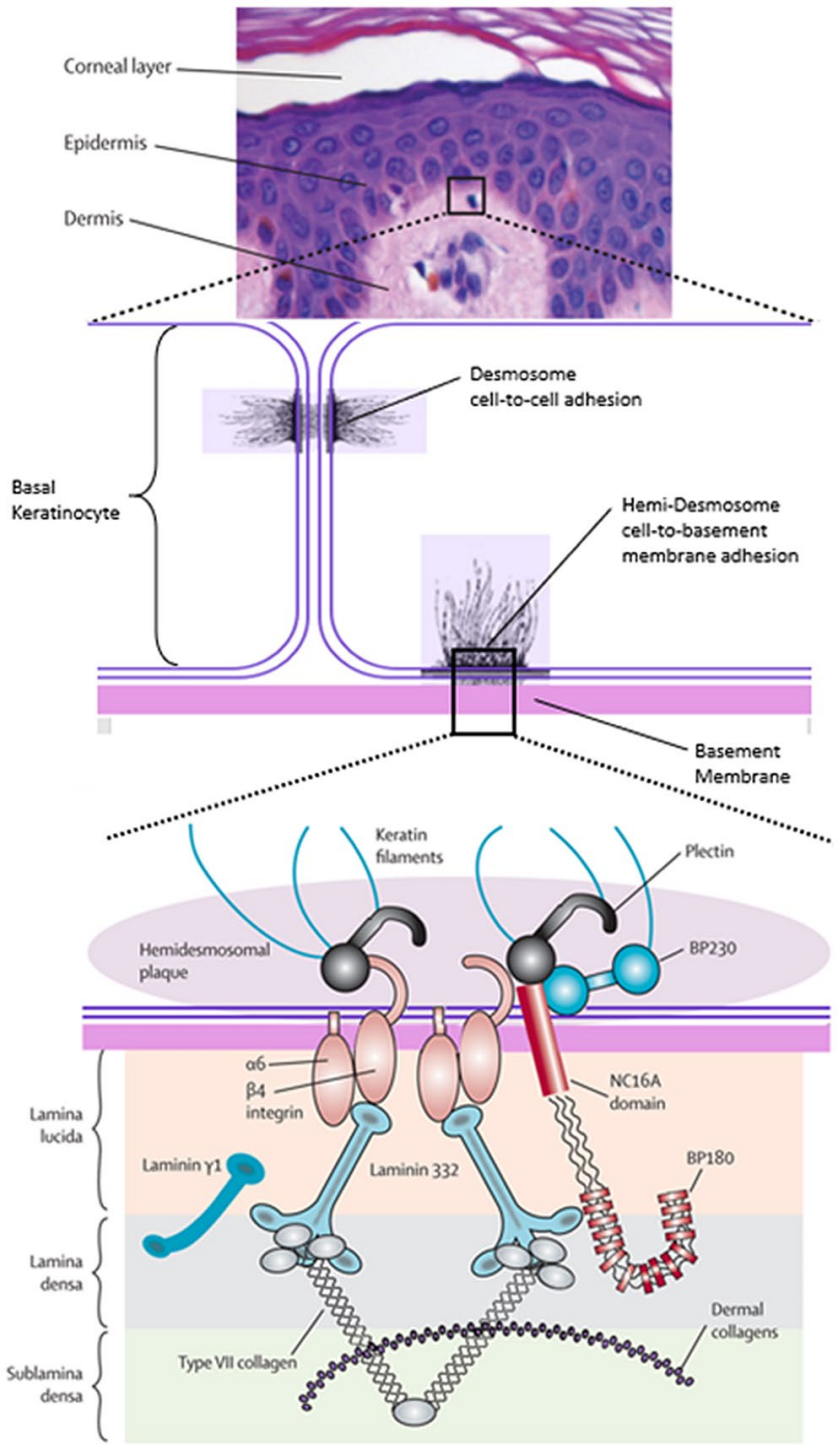
Schematic diagram of the dermoepidermal junction. Only molecules that are targeted by autoantibodies in pemphigoid diseases are shown (modified from Schmidt and Zillikens with permission) [2].

MMP has both clinical and immunological heterogeneity underpinning its pathogenesis. MMP is characterised by the development of autoantibodies targeting the normal ultrastructural components of hemidesmosomes that function to tether the basal keratinocytes to the underlying basement membrane zone of the mucosa. There are several components of the hemidesmosomes that have been identified as target antigens, including BP230, BP180, α6β4, laminin 332 and type VII collagen (Figure 1). Although autoantibodies have been recognised against these target antigens, so far, only autoantibodies targeting laminin 332 and α6β4 have been definitively established as being pathogenic in animal and in vitro models, respectively [1, 3, 4, 5, 6].

The main role of serology at present is only as an adjunct to diagnosis [7]. In contrast to bullous pemphigoid, the sera from patients with MMP contain autoantibodies at low titres, and much is still unknown about the role of the antibodies in prognostication or disease monitoring [1, 7, 8]. There is particular interest in laminin antibodies as several studies have shown an association with malignancy, although this has not been a consistent finding [9, 10, 11].

Regarding management, the 2021 European Academy of Dermatology and Venereology (EADV) Guidelines divides its recommendations between mild/moderate disease and severe disease, with the former defined as disease limited to the oral mucosa with or without skin involvement and severe defined as affecting extra‐oral/cutaneous sites. Rituximab was only recommended as second‐line treatment in severe disease or third‐line treatment in mild/moderate disease and with a warning about the risk of severe infections [7].

The largest study to date reporting the use of a rituximab in MMP was a retrospective case series of 109 severe/refractory MMP patients [12]. In this study, rituximab was administered as two 1 g doses, 14 days apart, repeated every 6 months, until complete remission or treatment failure. Immunosuppressive agents, such as azathioprine, were withdrawn on commencement of rituximab; however, immunomodulatory agents, such as dapsone, were continued until there was sustained remission off rituximab. Complete remission was obtained in 85% of patients, with a median of 2 cycles over 12 months of rituximab. These results are similar to those reported in a 2022 systematic review of 124 patients treated with rituximab for MMP in which 71% of patients achieved remission [13].

These studies clearly indicate a role for rituximab in the treatment of MMP; however, further research is required. In fact, clarification around the role of rituximab compared to standard immunosuppression was named as the top research priority in autoimmune bullous diseases (AIBD) in a recent aggregate survey of patients, carers, clinicians and researchers [14]. It remains unclear if there are biomarkers that can identify which patients will have relapse or refractory treatment requiring treatment intensification. Moreover, safety aspects, particularly in relation to infective risk, need to be addressed to instil confidence in prescribing clinicians intending to use rituximab.

## 2 Materials and Methods

This is a single‐centre, retrospective study of a cohort of MMP patients treated in a tertiary level, multidisciplinary, immunobullous clinic, between January 2015 and 2024, with a minimum follow‐up period of at least 6 months. The referral base consists of dental and medical practitioners based primarily in Sydney (population ~5 million). The clinic is staffed by oral medicine specialists and immunologists, and manages blistering skin and mucosal diseases with the aim of safely achieving disease remission with as little immunosuppression as practicable.

**FIGURE 2:**
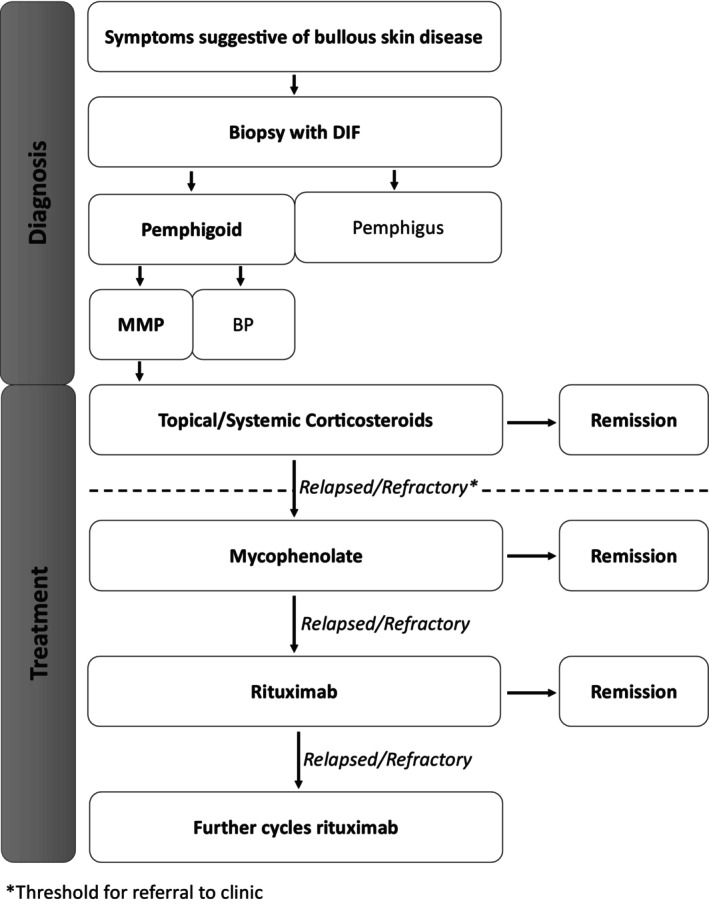
Oral immunobullous clinic workflow.

A schematic of the clinic's workflow is provided in Figure 2. As a prerequisite of referral to the clinic, all patients had a diagnosis of MMP, which was biopsy confirmed with either histopathological and/or direct immunofluorescence findings, and requirement for second‐line immunosuppression (i.e., mycophenolate) to replace prednisone if the dose was ≥ 10 mg/daily for disease control. Maximum tolerated dose of mycophenolate (mycophenolate mofetil up to 1500 mg bd or mycophenolate sodium up to 1080 mg bd) was used as a second‐line immunosuppressant, given our research demonstrating superior efficacy to azathioprine for pemphigus vulgaris and favourable safety profile [15]. Rituximab was used for those who had adverse effects or were intolerant to mycophenolate or, in the vast majority, who had either relapsed or were refractory to mycophenolate. The use of rituximab as a second‐line agent was largely based on our centre's success with pemphigus vulgaris [16], and in a smaller cohort with MMP. Rituximab was administered as two 1 g doses 14 days apart, with additional cycles in the event of relapse. We considered the use of rituximab to be a marker of refractory disease. In all patients receiving rituximab, their background immunosuppression, being mycophenolate and/or prednisolone, was ceased once significant clinical improvement had been observed.

Primary end point was achievement of complete or partial remission. This is in line with the 2015 consensus guidelines [17] with complete remission defined as no new or established lesions for at least 2 months and partial remission defined as presence of transient new lesions that heal within 1 week over the course of 2 months. Patients were secondarily evaluated in relation to relapse, complications of therapy and ability to wean off all other immunosuppression. Patients were also assessed in relation to serological and phenotypic biomarkers which may predict refractory or relapsed disease. Sustained remission was defined as a MMP Patient Disease Activity Index (MMPDAI) of 0, this being assessed every time on clinical review with no interval ulceration and no need for concomitant immunosuppression at the time of writing. The MMPDAI has been adapted from the validated PDAI score for pemphigus and was developed through consensus of an international group of experts [17]. A copy is available in the Supporting Information S1.

Data for patients were collected through review of the electronic medical records and patient assessments. At baseline, all patients had histological confirmation of the disease. In addition, circulating autoantibodies, for example, indirect immunofluorescence detection of skin autoantibodies, specifically skin basement membrane antibodies (SBMA), and more antigen‐specific markers BP180, BP230 and collagen VII were measured by ELISA immunoassays. Laminin 332 antibodies were also measured, although not necessarily at baseline, as this assay only became available for research use in October 2023, at the end of our study. At follow‐up, all patients were routinely evaluated for complications of therapy, including hypogammaglobulinaemia, infections and using COVID serology as a marker of sustained and maintained vaccine response.

Statistical analysis was computed with SPSS software (v29.0.1.0, IBM Corp). Quantitative variables were expressed as means and standard deviations. Differences between two groups were compared with *t* scores for continuous variables or by chi‐square/Fisher's exact tests for dichotomous variables.

## 3 Results

**FIGURE 3:**
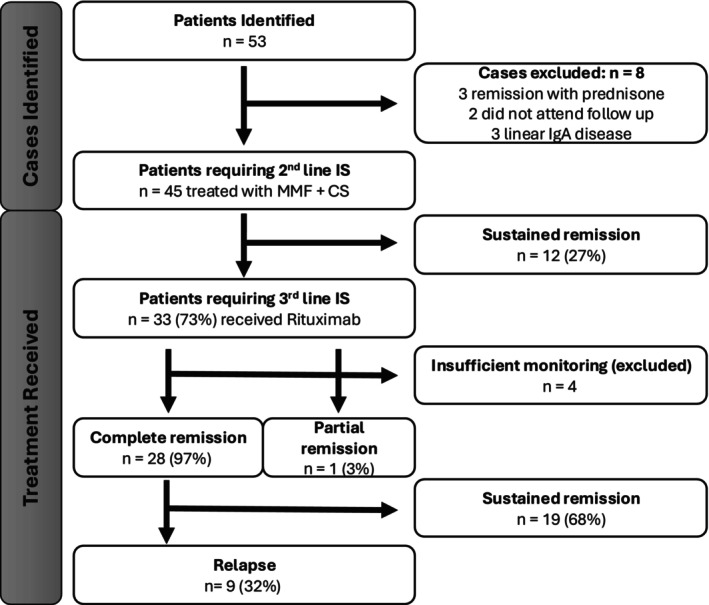
Study and patient flow. CS, corticosteroid; IS, immunosuppression; MMF, mycophenolate.

Fifty‐three patients were referred to the clinic with biopsy‐confirmed MMP. Eight were excluded as they did not meet the inclusion criteria (Figure 3). Forty‐five patients were included, of whom 12 (27%) achieved sustained remission with mycophenolate. Thirty‐three of the 45 patients (73%) required therapy intensification with rituximab. In one patient, intolerance to mycophenolate also contributed to this therapy change. Of the 33 patients who received rituximab, 28 (97%) achieved complete remission, which was sustained in 19 patients (68%) and 9 (32%) needing repeat treatment with rituximab. Four patients were excluded from analysis having been monitored for less than 6 months.

Patients were predominantly female (69%) and older on diagnosis, with a mean of 66 years. There was a slight majority of Caucasian patients (60%). Only 2 (4%) patients were prescribed gliptins on referral to the clinic. Seventeen (37%) patients had either solid organ (24%) or haematological (13%) malignancy. Of the solid organ malignancies, five were skin cancers, four of which were diagnosed prior to the development of MMP. The majority of patients, 37 (82%), had mild/moderate disease, that is, oral disease with or without cutaneous involvement. This likely reflects the pattern of referral to an oral immunobullous clinic where patients are referred early to reduce cumulative steroid burden. Overall circulating MMP‐associated autoantibody detection was low at baseline and at the time of inclusion in our case series with the following autoantibodies detected: SBMA in 7 (16%) of our patients, 9 with anti‐BP180/230 antibodies (20%), 0% with collagen VII antibodies and 2 (4%) with detectable circulating laminin 332 antibodies. Notably, of the two patients who were positive for laminin 332, neither had solid organ malignancy, although one patient did have myelofibrosis, a haematological malignancy. As shown in Table 1, there was no statistical difference in the clinical or serological characteristics in patients who were treated with mycophenolate versus those who required rituximab (*p* > 0.05).

**TABLE 1:** Baseline clinical and serological characteristics.*

	All	No rituximab^a^	Rituximab
	45	12 (27%)	33 (73%)
Demographics			
Gender (female)	31 (69%)	11 (92%)	20 (61%)
Age (years)	66 ± 13	68 ± 8	65 ± 14
Ethnicity (Caucasian)	27 (60%)	6 (50%)	21 (64%)
Gliptin (use on referral)	2 (4%)	1 (8%)	1 (3%)
Malignancy			
Solid organ	11 (24%)	2 (17%)	9 (27%)
Haematological	6 (13%)	1 (8%)	5 (15%)
Disease characteristics (severity of MMP)			
Mild/moderate	37 (82%)	11 (92%)	26 (79%)
Severe	8 (18%)	1 (8%)	7 (21%)
Antibodies (detected)			
SBMA	7 (16%)	1 (8%)	6 (18%)
BP 180/230	9 (20%)	2 (17%)	7 (21%)
Collagen VII	0	0	0
Laminin 332	2 (4%)	0	2 (6%)

^a^ People who achieved sustained remission on mycophenolate or prednisone.

* No statistical difference between groups (*p* > 0.05).

In patients who received rituximab, the mean time from diagnosis to rituximab therapy was 1288 days (±2909). The mean MMPDAI on commencement of therapy was 4.6 (±2.8), noting that in this disease the goal is no disease activity, that is, an MMP PDAI score of 0. There was no difference in baseline MMPDAI between patients who achieved sustained remission and those who relapsed. Patients required a low number of rituximab courses with a mean of 1.6 (±1). The highest number of rituximab courses was 6, throughout which the patient continued to attain complete remission with each course of treatment. Of the patients who did relapse after rituximab therapy, there was a long duration to first relapse with a mean of 615 (±226) days. As shown in Table 2, clinical and serological characteristics were not statistically significant (*p* > 0.05) between patients who relapsed after receiving rituximab therapy and those who did not, albeit this may be confounded by the low patient numbers.

**TABLE 2:** Clinical and serological characteristics of patients who received rituximab.*

	Sustained remission	Relapse
	19 (68%)	9 (32%)
Demographics		
Gender (female)	13 (68%)	4 (44%)
Age (years)	66 ± 12	61 ± 20
Ethnicity (Caucasian)	12 (63%)	5 (55%)
Gliptin (use on diagnosis)	1 (5%)	0 (0%)
Malignancy		
Solid organ	4 (21%)	3 (21%)
Haematological	3 (15.8%)	2 (22%)
Disease characteristics (severity of MMP)		
Mild/moderate	15 (79%)	7 (78%)
Severe	4 (21%)	2 (22%)
MMPDAI on rituximab commencement	4.6 ± 2.6	4.8 ± 2.8
Antibodies (detected)		
SBMA	4 (21%)	2 (22%)
BP 180/230	3 (16%)	2 (22%)
Collagen VII	0 (0%)	0 (0%)

* No statistical difference between groups (*p* > 0.05).

In relation to safety data collected, 14 (32%) patients developed hypogammaglobulinaemia, of whom 4 (9%) required ongoing treatment with intravenous immunoglobulin (IVIG). This included 5 (42%) patients receiving mycophenolate and 9 (28%) patients after receiving rituximab (*p* > 0.05). For the patients who received rituximab, there was a trend for a higher number of cycles of rituximab being associated with the development of hypogammaglobulinaemia (1.3 vs. 2.1), but this was not statistically significant (*p* = 0.06).

Eleven (24%) patients completed treatment in the pre‐COVID era. All patients treated during the COVID pandemic, who received at least two vaccines, demonstrated a serological vaccine response post treatment with immunosuppression (either mycophenolate or rituximab). No patient had a severe COVID infection. Of the patients who received mycophenolate, 2 (17%) had a mild COVID infection and 1 (8%) had a moderate COVID infection. Of the patients who received rituximab, 15 (48%) had a mild COVID infection and 1 (3%) had a moderate infection. There was no statistically significant difference between these groups, although more patients who received mycophenolate completed treatment pre‐COVID (*p* < 0.01).

## 4 Discussion

MMP is a classical immune disease mediated by autoantibodies against components of the basement membrane zone. Interestingly, despite multiple different self‐antigen targets identified, the clinical features and response to treatment do not differ with this pathogenic variation. Rituximab selectively induces B‐cell lysis by targeting CD20 expressed on the surface of B cells and thereby prevents the differentiation of B cells to become plasma cells able to produce the pathogenic autoantibodies. With no pathogenic autoantibodies present, the patients' MMP remits. Remarkably, with recovery of the B cells, usually in 6 months or more, there is often a ‘rebooting’ of the patients' immune system and patients do not redevelop symptoms. This phenomenon partly explains the durable remission rates seen in patients with MMP treated with rituximab and as seen in our cohort. Unsurprisingly, given this mechanistic link, patients, clinicians and researchers alike have deemed the clarification of the role of rituximab as a top research priority [14].

In our cohort, rituximab was an effective treatment for mycophenolate refractory MMP. In patients who received rituximab, 97% achieved complete remission after one course of rituximab and, of these patients, 68% had a durable remission. This is higher than what is reported in the literature, with larger studies reporting complete remission rates between 71% and 85% [12, 13, 18, 19]. There are a number of explanations for this; firstly, our cohort had more anatomically localised disease (oral and/or ocular) than other case series, and therefore, overall they had early, less severe disease, reflective of the referral bias of our oral immunobullous clinic. Secondly, our treatment paradigm was developed prior to the 2021 EADV Guidelines, and thus, rituximab was used early in the course of the patients' disease [7].

Treatment success in our cohort was further evidenced by the long duration of remission, even in those who did eventually relapse. The mean time to relapse was 1.7 years. In contrast, in a case series of 109 patients, 59% who achieved complete remission with rituximab did so for a median of 25 months [12]. This prolonged time to relapse in our patients may reflect the factors as outlined above.

There is significant concern regarding the risk of serious infections in patients treated with rituximab. In a 2020 study, patients with AIBD treated with rituximab had a fivefold higher incidence of COVID infections and were consequently at a higher risk of death [20]. Reassuringly, rituximab was well tolerated by patients in our study, which may reflect the rapid development of preventative and treatment options available for COVID. In our study, no patient in either treatment group had serious sequelae of COVID. Our patients had high uptake of COVID vaccines and, despite the use of immunosuppression, had serological evidence of vaccine response. Patients who had rituximab were not more likely to develop hypogammaglobulinaemia compared to those receiving mycophenolate. This may reflect the low number of cycles of rituximab required, especially when rituximab is used as an early, adjunctive immunosuppressant. Furthermore, in these patients, background immunosuppression was always ceased.

Given rituximab is so effective in the treatment of refractory MMP, there is the question as to whether there are predictors of which subtype of patients would benefit from receiving rituximab earlier. Recent literature has proposed several clinical and serological markers in MMP that may predict a more refractory phenotype; however, these results have not been uniform [12, 13, 18, 19, 21]. For example, a recent 2023 study purports that clinical features such as number/location of sites affected and serological features, such as dermal antibodies (detected by salt split mucosa on direct or indirect immunofluorescence) and the presence of laminin 332 antibodies, predict more refractory disease [19]. Conversely, these results were not demonstrated in a large case series published the year prior [12]. In our study, antibody detection rates were low and did not significantly predict refractory disease. Similarly, patients' baseline clinical characteristics did not predict refractory disease.

As demonstrated, rituximab usually needs to be given only as a single course. The use of limited cycles of rituximab has additional characteristics to recommend it as potential first‐line therapy in that, unlike other immunosuppressant agents, it does not appear to increase patients' risk for cutaneous malignancies [22]. This is a particular concern given the incidence of MMP occurs in an older cohort and, in Australia, within this older cohort are high rates of cutaneous malignancy [23]. Additionally, when used upfront, it will reduce the cumulative dose of oral steroids needed in elderly patients where steroid‐related complications are of considerable significance.

Our study has several limitations. Firstly, the patients had variable follow‐up periods. This may create bias, for example, in the relapse rates for patients followed for longer time periods. Secondly, due to the limited availability of antibody testing at our institution, laminin 332 antibodies were not tested at baseline, and therefore, the use of immunosuppression may have reduced detection rates. Although it is worth noting that overall circulating antibodies are low for MMP, particularly in relation to its counterpart, bullous pemphigoid [1, 8]. There may be several reasons for this, including the sequestration of autoantibodies at the site of disease (mucous membranes), the disease‐causing epitope not being adequately represented on the commercial ELISA substrate and patients being tested at different stages of disease activity. Finally, we acknowledge that there is emerging evidence for other therapeutic options, such as IVIG, which we did not utilise [24]. Our clinic workflow reflects local success using rituximab for pemphigus vulgaris [16] and lower efficacy of IVIG in select MMP patients with severe mucocutaneous disease in which it was trialled.

There are still many unanswered questions. Our cohort, despite being refractory, had relatively localised disease that responded well to rituximab. This raises the question if there is a role for earlier utilisation of rituximab, even in patients traditionally perceived to have only benign disease. Ideally, there would also be an easily measurable biomarker to guide clinicians; however, our study highlights that current antibody assays may not be able to adequately assist in this decision. As such, novel methods for biomarker detection need to be developed. A recent paper exploring conjunctival transcriptomics suggests one way forward [25]. Our group is now prospectively biobanking all immunobullous patients to determine if there are unique immunological signatures that would aid in risk‐stratifying patients and tailoring therapy.

In summary, rituximab is a safe and efficacious option for patients with refractory MMP, with more than 97% of patients in our study able to attain a complete remission after only one course. Furthermore, our study established that there is a role for this therapy even in patients with relatively localised disease. In light of this, we are undertaking research utilising rituximab as initial first‐line therapy for MMP. At present, there remains no reliable biomarker to predict which patients may require this therapy, and further research into establishing unique immunophenotype or ‘omic’ signatures for each individual that can tailor targeted therapy is required.

## Author Contributions

B.J.: acquisition of data, analysis, writing manuscript. M.S.: conception, acquisition of data and interpretation, revision of manuscript. E.A., E.S., S.R., D.J.: acquisition of data, revision of manuscript. D.C., S.C., D.M.: acquisition of data, approval of final manuscript. M.W.L.: conception and design, acquisition of data, interpretation of data, revision/drafting of manuscript.

## Ethics Statement

HREC Reference No. 2019/ETH02153. Approval date—26/07/2016. Expiry date—31/12/2025.

## Conflicts of Interest

The authors declare no conflicts of interest.

## Supporting information


Data S1.

## Data Availability

The data that support the findings of this study are available from the corresponding author upon reasonable request.
