# Supplementary material for: Rituximab for the Management of an Australian Cohort of Treatment Refractory Mucous Membrane Pemphigoid
Source: Australas J Dermatol. 2025 May 16;66(5):e271–8. doi: 10.1111/ajd.14523 (PMC12334813; doi:10.1111/ajd.14523)
Supplement: Supplementary file 1 — Data S1. [file AJD-66-e271-s001.docx]

**Supplementary text**

| **Infectious Diseases** | | | HIV |
| --- | --- | --- | --- |
|  |  |  | HCV |
|  |  |  | Hepatitis B core antigen |
|  |  |  | IGRA (for tuberculosis) |
|  |  |  | Strongyloidiasis antibodies |
|  |  |  | COVID19 Antibodies* |
|  |  |  | COVID19 Spike Protein Detection* |
| **Haematology** | | | Full Blood Count (FBC) |
|  |  |  | T and B-cell Quantification |
|  |  |  | Serum Immunoglobulin Levels  (IgA, IgG, and IgM) |
| **Serum Biochemistry** | | | Electrolytes |
|  |  |  | Urea/Creatinine Levels/eGFR |
| **Liver Function Tests** | | | Bilirubin Total |
|  |  |  | Total Protein |
|  |  |  | Albumin |
|  |  |  | Total Globulin |
|  |  |  | ALT |
|  |  |  | AST |
|  |  |  | GGT |
|  |  |  | ALP |
| **Glucose** | | | HbA1c |
|  |  |  | BSL |
| **Indirect Immuno-fluorescence (IIF)** | | | SBMA |
|  |  |  | BP 180 |
|  |  |  | BP 230 |
|  |  |  | Collagen VII |
| **Vitamin D** | | | 25 Hydroxy Vitamin D |
| *Abbreviations* | | | |
| *HIV* |  | *human immunodeficiency virus* | |
| *HCV* |  | *hepatitis C* | |
| *eGFR* |  | *estimated glomerular filtration rate* | |
| *ALT* |  | *alanine transaminase* | |
| *AST* |  | *aspartate aminotransferase* | |
| *GGT* |  | *gamma-glutamyl transferase* | |
| *ALP* |  | *alkaline phosphatase* | |
| *HbA1c* |  | *glycosylated hemoglobulin* | |
| *BSL* |  | *blood sugar levels* | |
| *SBMA* |  | *Skin basement membrane antibodies* | |

*Only performed on patients treated post 2019

**Table:** Baseline blood investigations for patients accepted to clinic.


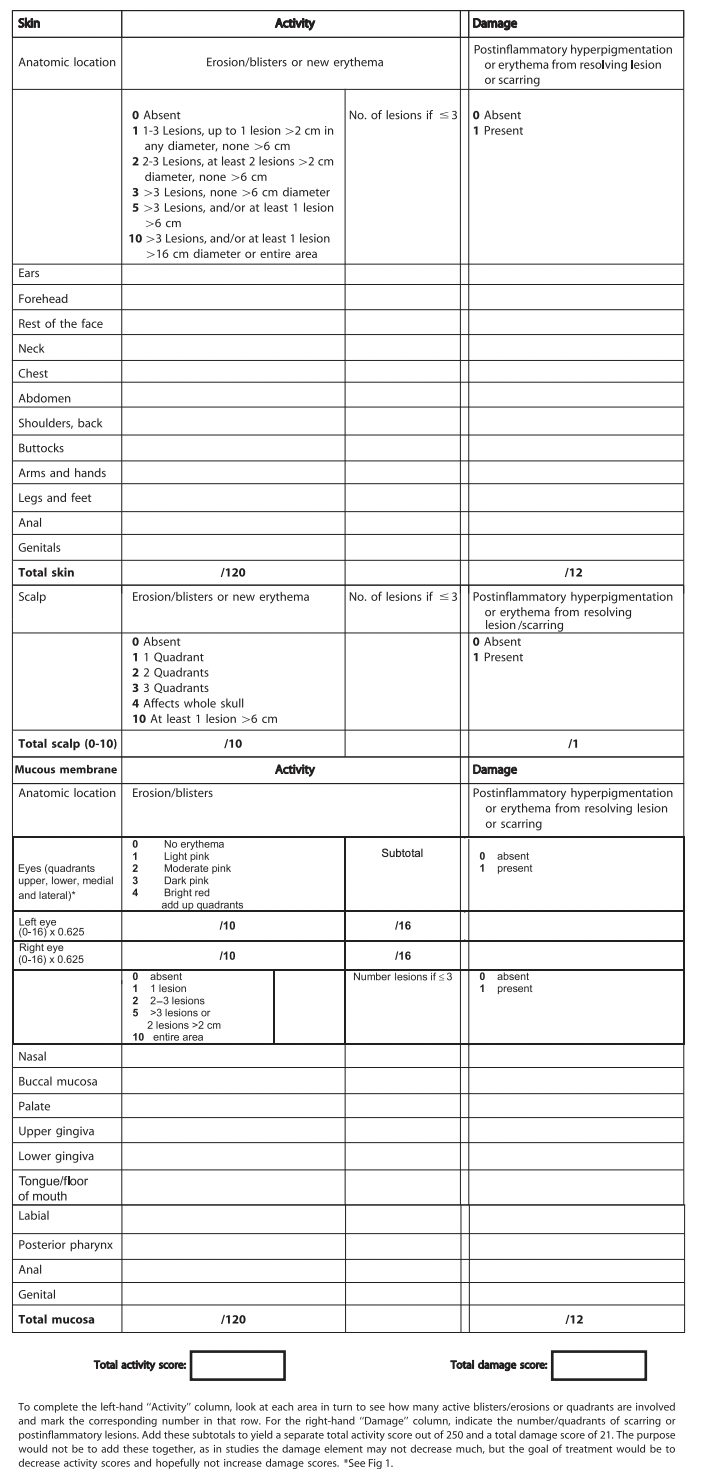


**Figure:** MMP Patient Disease Activity Index (MMPDAI) (17)
